# Antihypertensiva in der Psychiatrie: Verordnungsverhalten und Interaktionspotenzial

**DOI:** 10.1007/s00115-020-00996-9

**Published:** 2020-09-23

**Authors:** Katharina Endres, Ernst Schiller, Ekkehard Haen

**Affiliations:** 1 Institut AGATE gGmbH Pentling, Pentling, Deutschland; 2 Klinische Pharmakologie, Lehrstuhl für Pharmakologie und Toxikologie, Universität Regensburg, Universitätsstr. 31, 93053 Regensburg, Deutschland; 3 Rechenzentrum, Universität Regensburg, Universitätsstr. 31, 93053 Regensburg, Deutschland

**Keywords:** Pharmakoepidemiologie, Psychiatrie, Antihypertensiva, Arzneimittelinteraktionen, Hypertonie/Arzneimitteltherapie, Pharmacoepidemiology, Psychiatry, Antihypertensive agents, Drug interactions, Hypertension/drug therapy

## Abstract

**Hintergrund:**

Psychisch Kranke haben ein erhöhtes kardiovaskuläres Mortalitätsrisiko. Zur Prophylaxe und Therapie von Herz-Kreislauf-Erkrankungen werden insbesondere Antihypertensiva eingesetzt. In Kombination mit Psychopharmaka resultiert ein Interaktionspotenzial, wodurch das Erreichen therapeutischer Ziele beeinträchtigt werden kann.

**Ziel:**

Das Verordnungsverhalten bei Antihypertensiva in psychiatrischen Kliniken und Praxen im deutschsprachigen Raum sowie das Interaktionspotenzial mit Psychopharmaka soll untersucht werden.

**Methoden:**

Es erfolgte eine Auswertung der AGATE-„Stichtags“-Datenbank, welche anonym die Patientendaten Alter, Geschlecht, psychiatrische Hauptdiagnose sowie die verordneten Handelspräparate enthält. Die Auswertung der möglichen Interaktionen erfolgte mit PSIAC.

**Ergebnisse:**

Zwischen 01.01.2012 und 31.12.2016 wiesen 27 % aller 21.980 erfassten Patienten eine Verordnung für mindestens ein Antihypertensivum auf, wobei der Anteil mit dem Alter auf 72 % bei den über 80-Jährigen anstieg. 48 % der antihypertensiv Behandelten erhielten eine blutdrucksenkende Monotherapie. Mit dem Alter stieg die Bedeutung der antihypertensiven Kombinationstherapie. Insgesamt wurden den Patienten im Median 7 Wirkstoffe verordnet, wodurch mathematisch 21 Interaktionen resultieren. Durch eine gleichzeitige Gabe von Psychopharmaka und Blutdrucksenkern kann es vor allem zu einem erhöhten Risiko für Hypotonie, unzureichende Blutdrucksenkung oder QTc-Zeitverlängerung kommen.

**Diskussion:**

Antihypertensiva haben einen hohen Stellenwert bei der Behandlung psychiatrischer Patienten. Eine Interaktionsprüfung sollte durchgeführt werden, wenn die Pharmakotherapie ergänzt oder verändert werden soll. Allenfalls sollten Maßnahmen zur Verbesserung der Arzneimitteltherapiesicherheit erwogen werden.

## Abstract (en)

**Background:**

Patients with mental illnesses are at increased risk of cardiovascular disease-related mortality. Antihypertensive drugs are used particularly for the prophylaxis and treatment of cardiovascular diseases. In combination with psychiatric medication there is a potential for interaction, which can impair the achievement of therapeutic goals.

**Objective:**

The prescription behavior for antihypertensive drugs in psychiatric clinics and practices in German-speaking countries as well as the interaction potential with psychotropic drugs were investigated.

**Methods:**

The AGATE “index day” database, which anonymously stores patient data on age, sex, psychiatric main diagnosis as well as the prescribed commercial preparations collected in AGATE institutions (hospitals and community practitioners), was analyzed. The possible interactions were analyzed with PSIAC.

**Results:**

Between 1 January 2012 and 31 December 2016, 27% of all 21,980 patients recorded had a prescription for at least 1 antihypertensive drug, rising to 72% among patients over 80 years old. Of the patients treated with antihypertensive drugs, 48% received antihypertensive monotherapy. The importance of antihypertensive drug combination treatment increased with age. A median of 7 active substances were prescribed to the patients which mathematically results in 21 interactions. The simultaneous administration of psychotropic and antihypertensive drugs can result in an increased risk of hypotension, insufficient blood pressure reduction or QTc prolongation.

**Conclusion:**

Antihypertensive drugs are important in the treatment of psychiatric patients. An interaction test should be performed if pharmacotherapy is to be supplemented or changed. Where appropriate, measures to improve drug treatment safety should be considered.

Kardiovaskuläre Erkrankungen zählen zu den Hauptursachen für eine verminderte Lebenserwartung bei psychiatrischen Patienten. Zur medikamentösen Prävention und Therapie von Herz-Kreislauf-Krankheiten werden insbesondere Antihypertensiva eingesetzt. Diese Medikation ergänzt bei psychisch Kranken häufig die Psychopharmakotherapie und Wechselwirkungen werden möglich, die therapeutischen Zielen entgegenwirken. Das Verordnungsverhalten bei Blutdrucksenkern in psychiatrischen Kliniken und Praxen sowie das resultierende Interaktionspotenzial mit Psychopharmaka wird in diesem Beitrag dargestellt.

Die Lebenserwartung psychiatrischer Patienten ist ca. 10 Jahre geringer als die der Allgemeinbevölkerung, was meist auf Herz-Kreislauf-Erkrankungen zurückzuführen ist [15, 16, 28]. Zur Prävention und Therapie kardiovaskulärer Erkrankungen werden neben lebensstiländernden Maßnahmen vor allem Antihypertensiva eingesetzt [11, 13, 19, 20, 30]. Trotz der verschiedenen Optionen gelingt es häufig nicht, die kardiovaskulären Risikofaktoren der Patienten adäquat zu beeinflussen. Für Patienten mit psychischen Erkrankungen stellt bereits das Führen eines gesunden Lebensstils eine Hürde dar. Einige Studien zeigen, dass psychisch Kranke im Vergleich zur Allgemeinbevölkerung eher zu körperlicher Inaktivität, ungesunder Ernährung, Adipositas, metabolischem Syndrom, Alkoholkonsum bzw. -abhängigkeit und Rauchen neigen [4, 6, 18, 27]. Auch die Psychopharmakotherapie kann einen negativen Einfluss auf verschiedene kardiovaskuläre Risikofaktoren haben [26, 29]. Umso wichtiger ist eine effektive Arzneimitteltherapie mit Antihypertensiva. Da die Therapie mit Blutdrucksenkern bei psychiatrischen Patienten meist die Psychopharmakotherapie ergänzt, entsteht ein Interaktionspotenzial. Diese Arzneimittelwechselwirkungen können jedoch dem Ziel einer sicheren und wirksamen Pharmakotherapie entgegenwirken. Es stellt sich die Frage, welcher Anteil der psychiatrischen Patienten je nach Alter mit mindestens einem Antihypertensivum behandelt wird und damit potenziell einer klinisch relevanten Arzneimittelinteraktion mit Psychopharmaka ausgesetzt ist. Außerdem ist fraglich, wie viele Wirkstoffe den Patienten je nach Alter verordnet werden und welches Interaktionspotenzial daraus mathematisch resultiert. Nachdem eine blutdrucksenkende Polymedikation zur gewünschten Interaktion einer verstärkten Blutdrucksenkung führt, soll auch deren Anteil bestimmt werden. Bezüglich möglicher Interaktionen zwischen Psychopharmaka und Antihypertensiva stellt sich zunächst die Frage, welche Wirkstoffe am häufigsten bei psychiatrischen Patienten verordnet werden, die auch eine blutdrucksenkende Arzneimitteltherapie erhalten. Abschließend soll geklärt werden, mit welchen Wechselwirkungen zwischen den am häufigsten verordneten Psychopharmaka und Antihypertensiva gerechnet werden muss und wie diesen beizukommen ist.

## Methodik

Es erfolgte eine deskriptive Auswertung der AGATE-„Stichtags“-Datenbank, welche zentral die an den AGATE-Stichtagen in den Mitgliedskliniken und -praxen erhobenen Daten enthält. Die AGATE e. V. (www.amuep-agate.de) führt zweimal jährlich Stichtagserhebungen durch, an denen von allen am selben Tag behandelten Patienten anonym Alter, Geschlecht, psychiatrische Hauptdiagnose, verordnete Handelspräparate sowie deren Dosierungen dokumentiert werden [8]. Für die Datenbankauswertung bedurfte es laut lokaler Ethikkommission keiner Einwilligungserklärung. Es wurden alle vollständigen Datensätze erwachsener Patienten eingeschlossen, welche zwischen dem 01.01.2012 und 31.12.2016 erhoben wurden. Datensätze galten als vollständig, wenn Geschlecht, Alter und eine ICD-10-F-Hauptdiagnose angegeben wurden. Als Antihypertensiva wurden alle Arzneimittel aus den Hauptgruppen 17 (Antihypertonika), 27 (β-Rezeptoren‑, Kalziumkanalblocker und Hemmstoffe des Renin-Angiotensin-Aldosteron-Systems) und 36 (Diuretika) der Roten Liste definiert, anhand derer die Einteilung der Handelspräparate in der AGATE-„Stichtags“-Datenbank erfolgt. Zur Beschreibung der Stichprobe wurde die Gesamtanzahl und Anzahl vollständiger Datensätze bestimmt, ermittelt aus wie vielen Einrichtungen die eingeschlossenen Datensätze stammen, welches Alter die Patienten im Median aufwiesen, wie hoch der Frauenanteil war und wie sich die Patienten auf die ICD-10-F-Diagnosen verteilten. Es wurde die relative Häufigkeit der mit mindestens einem Antihypertensivum behandelten Patienten (PatAH) nach Altersklassen berechnet und das 95 %-Konfidenzintervall (95 %-KI) bestimmt. Der Median (x̃), das Minimum (x_min_) und Maximum (x_max_) sowie der Interquartilsbereich (Q_1_ [25 %-Perzentile]; Q_3_ [75 %-Perzentile]) der Anzahl verordneter Wirkstoffe aller Wirkstoffklassen wurden bei PatAH in allen Altersklassen bestimmt und daraus die Anzahl mathematisch möglicher Arzneimittelinteraktionen mit dem Binomialkoeffizienten „Wirkstoffanzahl über zwei“ berechnet. Die relative Häufigkeit der PatAH pro Altersklasse, die mit einer antihypertensiven Monotherapie, einer Kombination aus 2, 3 oder mehr als 3 Antihypertensiva behandelt wurden, wurde berechnet. Auch hier erfolgte die Bestimmung des 95 %-KI. Des Weiteren wurde ausgewertet, welche 25 Arzneistoffe am häufigsten bei PatAH (Top-25-Arzneistoffe) verordnet wurden und wie viele Antihypertensiva unter diesen Arzneistoffen waren. Die relative Häufigkeit, mit der diese Wirkstoffe bei PatAH verordnet wurden, wurde berechnet. Mit der Interaktionsdatenbank PSIAC wurden die möglichen Interaktionen zwischen den Psychopharmaka und Antihypertensiva, die unter den Top-25-Arzneistoffen waren, recherchiert und je nach klinischem Effekt und Handlungsempfehlung gruppiert. Es wurden lediglich Interaktionen berücksichtigt, die zu einem erhöhten Risiko für eine unerwünschte Arzneimittelwirkung führen können, für Risikopatienten kritisch sein können oder eine klinisch relevante Interaktion erwarten lassen. Unkritische Interaktionen und Kombinationen, bei denen bisher kein Interaktionsrisiko bekannt ist, wurden ausgeschlossen [10].

## Ergebnisse

### Beschreibung der Stichprobe

Im untersuchten Zeitraum wurden 31.125 Patientenfälle erhoben, von denen 21.980 in die Auswertung eingeschlossen werden konnten. Die Patientenfälle stammten aus 58 AGATE-Mitgliedseinrichtungen. Das Alter der Patienten bei Datenerhebung lag im Median bei 45 Jahren. Der Frauenanteil lag bei 45,2 %. In 33,5 % der Patientenfälle lag eine ICD-10-F3-, in 20,8 % eine -F2- und in 19,5 % eine -F1-Hauptdiagnose vor. Alle weiteren Patientenfälle verteilten sich auf die ICD-10-Diagnosen F0, F4, F5, F6, F7, F8 und F9.

### Anteil der antihypertensiv behandelten Patienten

**Figure 1:**
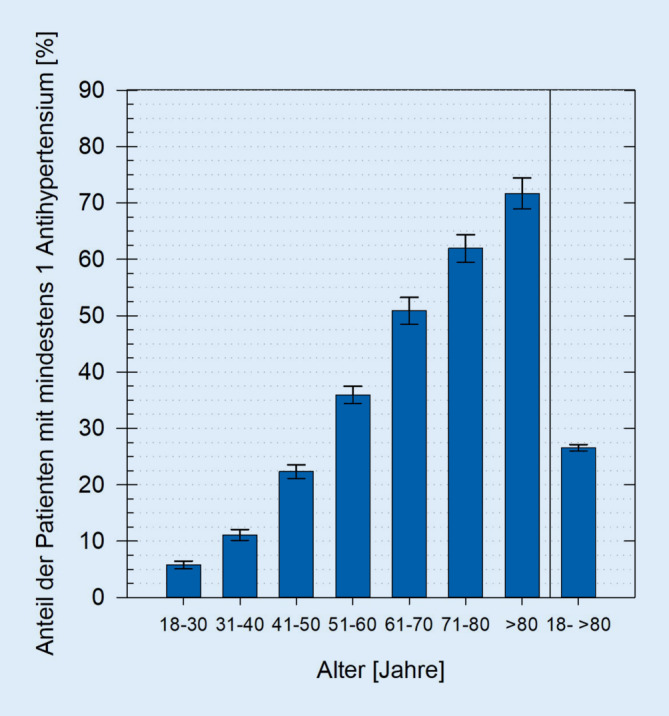

Von allen eingeschlossenen Patientendatensätzen lag in 27 % eine Verordnung für mindestens ein Antihypertensivum vor. War der Anteil der PatAH bei den 18- bis 30-Jährigen noch bei 6 %, so stieg er mit dem Alter stetig auf 72 % bei den über 80-Jährigen (Abb. 1).

### Polypharmazie und Interaktionspotenzial

**Figure 2:**
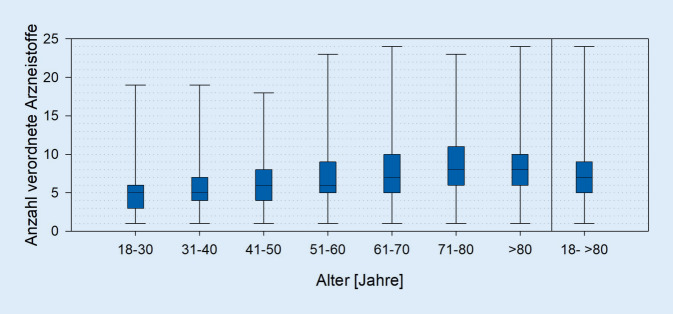

Im Median wurden den PatAH unabhängig vom Alter 7 Wirkstoffe (x_min_ = 1; x_max_ = 24; Interquartilsbereich: [5, 9]) verordnet, wodurch ein hohes Interaktionspotenzial besteht. Bei 5 verordneten Wirkstoffen ergeben sich 10, bei 7 Stoffen 21, bei 24 Arzneistoffen 276 mathematisch mögliche Wechselwirkungen. Mit zunehmendem Alter nahm der Median der Anzahl verordneter Wirkstoffe zu, wodurch gerade geriatrische Patienten einer hohen Zahl möglicher Arzneimittelinteraktionen ausgesetzt waren (Abb. 2).

### Bedeutung antihypertensiver Kombinationstherapien

**Figure 3:**
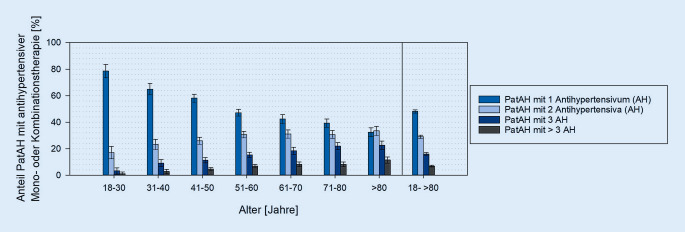

Insgesamt 48 % der antihypertensiv Behandelten erhielten eine Monotherapie mit Blutdrucksenkern. Eine Kombination aus 2 Antihypertensiva erhielten 29 %, eine Dreierkombination 16 % und mehr als 3 Blutdruckmittel erhielten 7 % der PatAH. Der Anteil der Patienten mit einer blutdrucksenkenden Monotherapie war bei 18- bis 30-Jährigen am größten und sank altersabhängig. Im Gegenzug wurden vermehrt blutdrucksenkende Kombinationstherapien verordnet (Abb. 3).

### Top-25-Arzneistoffe

Die 25 am häufigsten verordneten Arzneistoffe und deren relative Häufigkeit der Verordnung bei PatAH sind in Tab. 1 aufgeführt. Unter den Top-25-Arzneistoffen waren 7 Antihypertensiva.

**Table 1:** 

	Top-25-Arzneistoffe	(% PatAH)
1	Ramipril	37,42
2	Pantoprazol	34,37
3	Bisoprolol	25,05
4	Acetylsalicylsäure	22,85
5	Quetiapin	20,08
6	Metoprolol	19,31
7	Levothyroxin	19,21
8	Hydrochlorothiazid	17,90
9	Mirtazapin	16,22
10	Amlodipin	16,12
11	Simvastatin	15,92
12	Lorazepam	15,40
13	Torasemid	14,24
14	Venlafaxin	12,83
15	Risperidon	12,32
16	Metformin	11,50
17	Pipamperon	8,65
18	Kaliumchlorid	8,31
19	Thiamin (Vitamin B1)	8,07
20	Citalopram	7,37
21	Olanzapin	7,18
22	Valproinsäure	6,78
23	Sertralin	6,66
24	Melperon	6,58
25	Valsartan	6,53

*PatAH* mit mindestens einem Antihypertensivum behandelte Patienten

### Auswertung möglicher Interaktionen

Erweitert man eine Psychopharmakotherapie mit Antihypertensiva, ergeben sich nach einer Auswertung mit PSIAC 29 mögliche Wechselwirkungen. Die Interaktionen lassen sich anhand der erwarteten klinischen Effekte sowie der empfohlenen Maßnahmen zur Verbesserung der Arzneimitteltherapiesicherheit in 5 Gruppen zusammenfassen (Tab. 2).

**Table 2:** 

Effekt	Psychopharmaka	Antihypertensiva	Handlungsempfehlungen, wenn eine Kombination erforderlich ist
Verstärkte Blutdrucksenkung, orthostatische Hypotonie	Quetiapin Risperidon	Amlodipin Bisoprolol Hydrochlorothiazid Metoprolol Ramipril Torasemid Valsartan	Blutdruckkontrolle (Blutdruckmessung und Schellong-Test), Dosisanpassung der Antihypertensiva erwägen, Antipsychotika langsam eindosieren
Verminderte Blutdrucksenkung	Venlafaxin	Amlodipin Bisoprolol Hydrochlorothiazid Metoprolol Ramipril Valsartan	Blutdruckkontrolle, Dosisanpassung der Antihypertensiva erwägen
Additive QTc-Zeit-Verlängerung	Citalopram Melperon Mirtazapin Olanzapin Pipamperon Sertralin Venlafaxin	Torasemid	QTc-Zeit prüfen (>450 ms?), Serumkalium- und Serummagnesiumspiegel hochnormal einstellen, weitere Risikofaktoren abklären
Erhöhtes Risiko für unerwünschte Wirkungen durch Torasemid	Melperon	Torasemid	Dosisanpassung von Torasemid erwägen
Erhöhtes Risiko für eine Bradykardie	Melperon	Metoprolol	EKG kontrollieren

*QTc-Zeit* Dauer des herzfrequenzkorrigierten QT-Intervalls, *EKG*  Elektrokardiogramm

## Diskussion

### Anteil antihypertensiv behandelter Patienten

Gut ein Viertel der Patienten wurde mit mindestens einem Antihypertensivum behandelt und war damit potenziell einer Kombinationstherapie aus Psychopharmaka und Blutdrucksenkern ausgesetzt. Dieser Anteil nahm altersabhängig zu, was nicht überraschte, da Alter ein Risikofaktor zur Entwicklung kardiovaskulärer Erkrankungen ist [19]. Bei der Interpretation der Zahlen muss berücksichtigt werden, dass Blutdrucksenker, wie β‑Blocker oder auch der α_2_-Agonist Clonidin, in der Psychiatrie auch zur Behandlung von z. B. lithiuminduziertem Tremor, Akathisie durch Antipsychotika oder vegetativen Symptomen des Alkoholentzugssyndroms eingesetzt werden [14, 17]. Auch kommt der Einsatz bei nichtpsychiatrischen Indikationen, wie portaler Hypertonie, Migräneprophylaxe oder essenziellem Tremor infrage [14]. Da lediglich die psychiatrischen Hauptdiagnosen erfasst wurden, kann durch die Zahlen nicht auf die Prävalenz kardiovaskulärer Erkrankungen bei psychiatrischen Patienten geschlossen werden. Auch lässt sich nicht ableiten, wie effektiv kardiovaskuläre Risikofaktoren durch die Therapie mit Antihypertensiva beeinflusst wurden.

### Bedeutung der Polypharmazie

In der allgemeinen deutschen Erwachsenenpopulation ist laut DEGS1-Studie die verschriebene Polypharmazie, definiert als Anwendung von mindestens 5 verordneten Präparaten innerhalb der letzten 7 Tage, von großer Bedeutung. Über 40 % der 70- bis 79-Jährigen waren in der DEGS1-Studie von einer verschriebenen Polymedikation betroffen [12]. Nachermittelte Daten der AGATE-„Stichtags“-Datenbank zeigen, dass 69 % der 71- bis 80-jährigen psychisch Kranken sowie 89 % der gleichaltrigen PatAH mit mindestens 5 Präparaten behandelt wurden. Psychiatrische Patienten sind demnach im Vergleich zur Allgemeinbevölkerung häufiger einer verordneten Polymedikation ausgesetzt. Da die Selbstmedikation bei der Stichtagserhebung nicht berücksichtigt wird, muss insgesamt von einer noch höheren Arzneimittelexposition ausgegangen werden. Betrachtet man die konkrete Anzahl verordneter Wirkstoffe und die daraus resultierende Anzahl möglicher Interaktionen, sind besonders betagte psychiatrische Patienten einem Risiko für Arzneimittelwechselwirkungen und einem damit einhergehenden Risiko für unerwünschte Arzneimittelwirkungen ausgesetzt. Die Zahl möglicher Interaktionen stellt jedoch nur die Anzahl mathematisch möglicher Wechselwirkungen dar. Eine Kombination von Wirkstoffen kann jedoch zu mehreren pharmakokinetischen und pharmakodynamischen Effekten führen, die von unterschiedlicher klinischer Relevanz sein können [7]. Teilweise können diese Interaktionen auch erwünscht sein, wie bei der Kombination mehrerer Antihypertensiva. Neben dem zunehmenden Risiko für Arzneimittelinteraktionen erhöht sich mit wachsender Anzahl verordneter Präparate auch die Wahrscheinlichkeit für Probleme bei der Therapietreue, wodurch das Erreichen der Therapieziele wiederum beeinträchtigt werden kann [2].

### Blutdrucksenkende Mono- und Kombinationstherapien

Eine Kombination mehrerer Antihypertensiva ist üblich bei der Behandlung von Bluthochdruck, koronarer Herzkrankheit oder Herzinsuffizienz. Es wird empfohlen bis zu 3 Wirkstoffe gleichzeitig einzusetzen, wobei bei resistenter Hypertonie noch mehr Blutdrucksenker kombiniert werden können [13, 20, 30]. Der hohe Einsatz antihypertensiver Kombinationstherapien bei psychiatrischen Patienten ist daher prinzipiell positiv zu bewerten und relativiert die Bedeutung der Polymedikation und des Interaktionspotenzials. Bezüglich möglicher Complianceprobleme durch die erforderliche Einnahme mehrerer Darreichungsformen wäre interessant, welche und in welchem Ausmaß Fixkombinationen bei psychisch Kranken verordnet wurden. Solch eine Auswertung ist mit der AGATE-„Stichtags“-Datenbank zum jetzigen Zeitpunkt aus technischen Gründen jedoch nicht möglich.

### Die am häufigsten verordneten Arzneistoffe

Unter den Top-25-Arzneistoffen bei PatAH waren 7 Antihypertensiva, die alle zu den 5 Hauptarzneistoffklassen zur Behandlung der Hypertonie zählen [30]. Am häufigsten wurden die Patienten mit Ramipril behandelt, das auch im ambulanten Bereich in den Jahren 2012 bis 2016 unter den Blutdrucksenkern am häufigsten eingesetzt wurde [21–25]. Neben den Antihypertensiva finden sich in der Auflistung noch Acetylsalicylsäure, Metformin und Simvastatin. Auch diese Wirkstoffe werden zur Prävention und Therapie von Herz-Kreislauf-Erkrankungen bzw. zur Verbesserung kardiovaskulärer Risikofaktoren eingesetzt [13, 19]. Unter den Top-25-Arzneistoffen finden sich, wie bei der Stichprobe zu erwarten war, auch viele Psychopharmaka. Mit Quetiapin, Mirtazapin, Venlafaxin, Pipamperon, Citalopram und Olanzapin können viele von ihnen zu einer Blutdrucksenkung oder orthostatischen Hypotonie führen [5]. Andererseits können die meisten der zuvor genannten Wirkstoffe sowie Risperidon und Valproinsäure zu Übergewicht, Dyslipidämie, Diabetes mellitus oder Hypertonie führen. Diese Arzneistoffe üben also auch einen negativen Einfluss auf das kardiovaskuläre Risiko aus, was die adäquate Behandlung der Patienten erschwert [1].

### Mögliche Arzneimittelinteraktionen

Nach der Prüfung möglicher Wechselwirkungen zwischen Psychopharmaka und Antihypertensiva mit der Interaktionsdatenbank PSIAC muss vor allem mit einer zu starken oder nur unzureichenden Blutdrucksenkung gerechnet werden. Bei Patienten, die Torasemid erhalten, muss außerdem berücksichtigt werden, dass Torasemid ein konditionelles Risiko für Torsade-de-pointes(TdP)-Tachykardien bzw. QTc-Zeit-Verlängerung besitzt. In Kombination mit vielen Psychopharmaka erhöht sich das Gesamtrisiko für diese kritische Nebenwirkung. Mit Melperon, das eine klinisch relevante CYP2D6-hemmende Wirkung aufweist, muss bei gleichzeitiger Anwendung von Torasemid oder Metoprolol mit erhöhten Wirkspiegeln, einer verstärkten Wirkung und einem höheren Risiko für Nebenwirkungen gerechnet werden, da beide Blutdrucksenker vorwiegend über dieses CYP-Isoenzym verstoffwechselt werden. Im Falle der Kombination von Torasemid mit Melperon gilt besonders das Risiko für QTc-Zeitverlängerung zu beachten, da beide Wirkstoffe das Risiko für diese Nebenwirkung erhöhen können. Bei einer gleichzeitigen Behandlung mit Metoprolol und Melperon wurde klinisch ein erhöhtes Risiko für Bradykardien beobachtet [10]. Um dieses Risiko zu senken, ist es neben der Überwachung des EKG auch möglich, Metoprolol gegen einen β‑Blocker auszutauschen, der in geringerem Umfang über die Leber metabolisiert wird. Infrage kommen beispielsweise Bisoprolol oder Atenolol [3]. Wie häufig die in Tab. 2 genannten Kombinationstherapien aus Psychopharmaka und Antihypertensiva tatsächlich vorkommen, kann aus technischen Gründen mit der AGATE-„Stichtags“-Datenbank nicht ausgewertet werden. Für die Praxis lässt sich dennoch festhalten, dass sich die meisten der genannten Interaktionen gut überwachen und regeln, zum Teil auch vermeiden lassen, sofern sie erkannt werden. Für eine effiziente Überprüfung möglicher Wechselwirkungen empfiehlt sich die Anwendung von Interaktionsdatenbanken, die auch Handlungsempfehlungen geben [9].

## Fazit für die Praxis

- Antihypertensiva haben einen hohen Stellenwert bei der Behandlung psychiatrischer Patienten.
- Die blutdrucksenkende Pharmakotherapie erweitert in den häufigsten Fällen die Polymedikation um 1 bis 2 Arzneistoffe und kann zu vielfältigen Wechselwirkungen führen.
- Insbesondere eine adäquate Blutdruckeinstellung kann durch diese Interaktionen erschwert werden.
- Vor Erweiterung oder Umstellung der Psychopharmakotherapie sollte ein Interaktionscheck durchgeführt werden, um mögliche Risiken abschätzen und gegebenenfalls senken zu können.
